# Correlation of Indoleamine-2,3-Dioxygenase and Chronic Kidney Disease: A Pilot Study

**DOI:** 10.1155/2021/8132569

**Published:** 2021-01-06

**Authors:** Binbin Pan, Feng Zhang, Jian Sun, Dawei Chen, Wenjuan Huang, Hao Zhang, Changchun Cao, Xin Wan

**Affiliations:** ^1^Department of Nephrology, Nanjing First Hospital, Nanjing Medical University, Nanjing, China; ^2^Department of Nephrology, Sir Run Run Hospital, Nanjing Medical University, Nanjing, China

## Abstract

**Objective:**

To explore the correlation of indoleamine-2,3-dioxygenase (IDO) and chronic kidney disease (CKD).

**Methods:**

A total of 154 CKD patients and 42 non-CKD patients were recruited. Patients were grouped into ACR1~ACR3 (<30 mg/g, 30-300 mg/g, and >300 mg/g). Biomarkers in different groups were compared by ANOVA. Correlation was calculated by Pearson or Spearman analysis and binary logistic regression. The ROC curve was also performed.

**Results:**

The levels of albumin, serum creatinine (sCr), and IDO in non-CKD patients were significantly different from those in CKD3-CKD5 stages (*p* < 0.05). IDO was correlated with age, proteinuria, ACR, and eGFR (*p* < 0.01). After adjusting for CKD-related indices, ln(IDO) was an independent risk factor for CKD (3.48, *p* < 0.05). The analysis of ROC curve revealed a best cut­off for IDO was 0.0466 and yielded a sensitivity of 83.8% and a specificity of 75%. Hemoglobin, total protein, and albumin in the ACR1 group were significantly higher than those in the ACR2 and ACR3 groups (*p* < 0.01), while sCr and IDO levels were significantly lower than those in the ACR2 and ACR3 groups (*p* < 0.01 or *p* < 0.05). After adjusting for CKD-related indices, ln(IDO) was still an independent risk factor for ACR (OR = 2.7, *p* < 0.05). The analysis of ROC curve revealed a best cut­off for IDO was 0.075 and yielded a sensitivity of 71.9% and a specificity of 72.2%.

**Conclusion:**

IDO may be a promising biomarker to predict CKD and assess kidney function.

## 1. Introduction

Chronic kidney disease (CKD) is a worldwide epidemic disease with more than 10.8% prevalence in China [1]. The final common pathology of CKD is kidney fibrosis [2, 3]. The severity of CKD is in accordance with the degree of fibrosis level. However, no data to date are available to cure CKD. Therefore, early diagnosis and management of CKD is a common sense for nephrologists. Clinically, serum creatinine (sCr), cystatin C, and *β*2-microglobulin are used commonly for assessing the severity of CKD. Nevertheless, elevations of the above markers suggest that a decrease of kidney functions is rather severe. Hence, exploring novel markers to assess CKD is indispensable.

Tryptophan (Trp) can be catalyzed by indoleamine-2,3-dioxygenase (IDO) into kynurenine (Kyn), kynurenic acid, and quinolinic acid [4, 5]. Therefore, IDO activity is often assessed by the ratio of Kyn and Trp [6–8]. A previous study in systemic sclerosis patients showed elevated levels of Kyn and decreased levels of Trp compared with limited cutaneous patients or healthy controls [8], suggesting increased levels of IDO. IDO was also significantly expressed in cortical and medulla tubules of the UUO mice [9] indicating IDO may be associated with kidney fibrosis. Diabetic nephropathy study also showed IDO activity was parallel with the severity of CKD and negatively correlated with estimated glomerular filtration rate (eGFR) [10]. Furthermore, induced expression of IDO could cause cell arrest in G0/G1 via Trp depletion resulting in cardiac fibrosis which could be ameliorated by IDO inhibitor, 1-methyltryptophan [11]. Therefore, IDO is a potential biomarker for assessing tissue fibrosis and CKD.

Although previous studies have focused on the correlation between kidney function and IDO, little is known about the predicting function of IDO on the occurrence of CKD. In the present study, we explored the correlation of IDO with CKD-related indices and the predictive ability of IDO on CKD and albuminuria and creatinine ratio (ACR).

## 2. Materials and Methods

A total of 196 patients were recruited from December 2018 to May 2020 in Nanjing First Hospital. We excluded subjects who had the following characteristics: younger than 18 years old, fever, pregnancy, heart failure, liver dysfunction, diabetic ketoacidosis, nonketotic hyperosmolar coma, diabetic lactic acidosis, taking *α*-ketoacid in the past four weeks, and acute kidney injury.

### 2.1. Laboratory Measure

Cystatin C, sCr, blood urea nitrogen (BUN), uric acid, *β*2-microglobin, serum albumin, calcium, phosphorus, serum lipid, and proteinuria were determined by the OLYMPUS AU5400 automatic biochemical analyzer (Olympus Corporation, Mishima, Japan). The calibrators for the enzymatic method were traceable to an isotope dilution mass spectrometric method for sCr using standard reference methods NIST SRM 967 [12]. ACR and D-dimer were determined via immunoturbidimetry. Plasma prothrombin time, activated partial prothrombin time, and plasma fibrinogen were measured by the coagulation method. Thyroid function was tested by using an electrochemiluminescence assay (Siemens, Centaur XP). Hemoglobin was measured by the Sysmex XT­1800i Automated Hematology System (Shanghai, China). The values of serum immunoglobulin and complements were tested by single immunodiffusion. C-reactive protein (CRP) was tested by electrochemiluminescence. Trp and Kyn of blood samples were determined by high-performance liquid chromatography-mass spectrometry. The level of IDO was calculated by the ratio of Kyn and Trp.

### 2.2. Definition of CKD

Different CKD stages were divided following the KIDGO guideline: stage 1, eGFR ≥ 90 ml/min/1.73m^2^; stage 2, 60 ml/min/1.73m^2^ ≤ eGFR < 90 ml/min/1.73m^2^; stage 3, 30 ml/min/1.73m^2^ ≤ eGFR < 60 ml/min/1.73m^2^; stage 4, 15 ml/min/1.73m^2^ ≤ eGFR < 30 ml/min/1.73m^2^; stage 5, eGFR < 15 ml/min/1.73m^2^. The Chronic Kidney Disease Epidemiology Collaboration (CKD-EPI) four-level race equation was employed to calculate eGFR [13, 14]. The specific CKD-EPI four-level race GFR estimation equation was as follows:

(1) $$\begin{array}{c} \text{eGFR}=\text{EXP}\left(\text{LN}\left(151\right)-0.328*\text{LN}\left(\text{sCr}/88.4/0.7\right)+\text{age}*\text{LN}\left(0.993\right)\right)\left(\text{If female and creatinine}<0.7\right)=\text{EXP}\left(\text{LN}\left(151\right)-1.210*\text{LN}\left(\text{sCr}/88.4/0.7\right)+\text{age}*\text{LN}\left(0.993\right)\right)\left(\text{If female and creatinine}\geq 0.7\right)=\text{EXP}\left(\text{LN}\left(149\right)-0.412*\text{LN}\left(\text{sCr}/88.4/0.9\right)+\text{age}*\text{LN}\left(0.993\right)\right)\left(\text{If male and creatinine}<0.9\right)=\text{EXP}\left(\text{LN}\left(149\right)-1.210*\text{LN}\left(\text{sCr}/88.4/0.9\right)+\text{age}*\text{LN}\left(0.993\right)\right)\left(\text{If male and creatinine}\geq 0.9\right). \end{array}$$

### 2.3. Statistical Analysis

Statistics analysis was performed by PASW 22.0 statistical software (SPSS Inc., Chicago, IL, USA). Data were expressed as mean ± SD. One-way ANOVA was used to compare means for continuous variables. The LSD method was used for continuous variables with homogeneous variances, and Dunnett's method was used for continuous data with uneven variances. The Pearson or Spearman correlation analysis was employed to determine the correlations between IDO and other indices. In addition, the binary logistic regression was employed to explore independent influence factors for CKD and ACR. The receiver operating characteristic (ROC) curve was built to evaluate the prediction of IDO on CKD and ACR. The best cutoff for ROC curve was calculated with Youden's index. *p* value < 0.05 was considered to be statistically significant.

## 3. Results

### 3.1. Clinical Characteristics of Patients (Table 1)

A total of 196 patients were recruited. There were 154 patients with CKD and 42 non-CKD patients. Among them, there were 3 cases in CKD1 and 6 cases in CKD2, respectively. Therefore, the accurate data of CKD1 and CKD2 were not listed. There were no significant differences in gender, prealbumin, free thyroxine, thyroid-stimulating hormone, complement C4, total cholesterol, low-density lipoprotein, high-density lipoprotein, glycosylated hemoglobin, immunoglobulin A, immunoglobulin M, immunoglobulin G, and CRP among non-CKD patients and CKD3 to CKD5 patients (*p* > 0.05). The levels of uric acid, albumin, cystatin C, *β*2-microglobulin, BUN, sCr, calcium, Kyn, and IDO in non-CKD patients were significantly different from those in CKD3-CKD5 stages (*p* < 0.05). There were significant differences in age, free triiodothyronine, hemoglobin, and serum phosphorus between non-CKD patients and CKD4 and CKD5 patients (*p* < 0.05). The levels of triglyceride and Trp in non-CKD patients were significantly higher than those in CKD5 patients (*p* < 0.05).

## 4. Correlations between IDO and Clinical Indices

**Figure 1:**
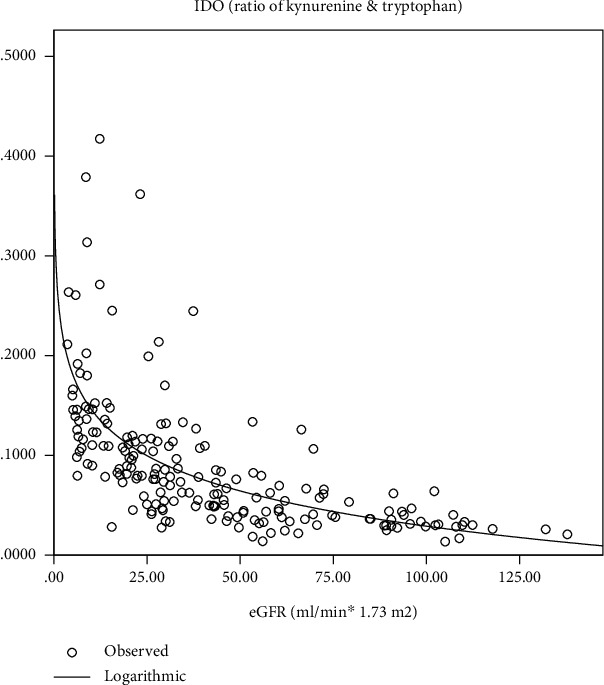
Estimated curve between IDO and eGFR.

There was a positive correlation between IDO and age, serum phosphorus, CRP, ACR, plasma prothrombin time, activated partial prothrombin time, fibrinogen, D-dimer, hypertension, and uric acid (*r* = 0.3, *p* < 0.01; *r* = 0.4, *p* < 0.01; *r* = 0.3, *p* < 0.01; *r* = 0.5, *p* < 0.01; *r* = 0.3, *p* < 0.01; *r* = 0.2, *p* < 0.01; *r* = 0.4, *p* < 0.01; *r* = 0.4, *p* < 0.01; *r* = 0.2, *p* < 0.01; *r* = 0.4, *p* < 0.01). There was a negative correlation between IDO and eGFR, albumin, serum calcium, and hemoglobin (*r* = −0.8, *p* < 0.01; *r* = −0.6, *p* < 0.01; *r* = −0.5, *p* < 0.01; *r* = −0.6, *p* < 0.01) (Table 2). As IDO had a high correlation coefficient with eGFR, an estimated curve between IDO and eGFR was performed (Figure 1). As IDO is not normal distribution data, logarithm was employed to transform IDO into ln(IDO) which is normal distribution data. Further, binary logistic regression analysis was performed with CKD or not as dependent variances. The result showed that ln(IDO) was an independent risk factor for CKD. After adjusting for age, ACR, hypertension, diabetes, coronary heart disease, cerebral infarction, and ln(IDO), age, ACR, and ln(IDO) were independent risk factors for CKD (OR = 1.07, *p* < 0.05; 1.14, *p* < 0.01; 3.48, *p* < 0.05) (Table 3).

### 4.1. The Efficiency of IDO to Predict CKD

**Figure 2:**
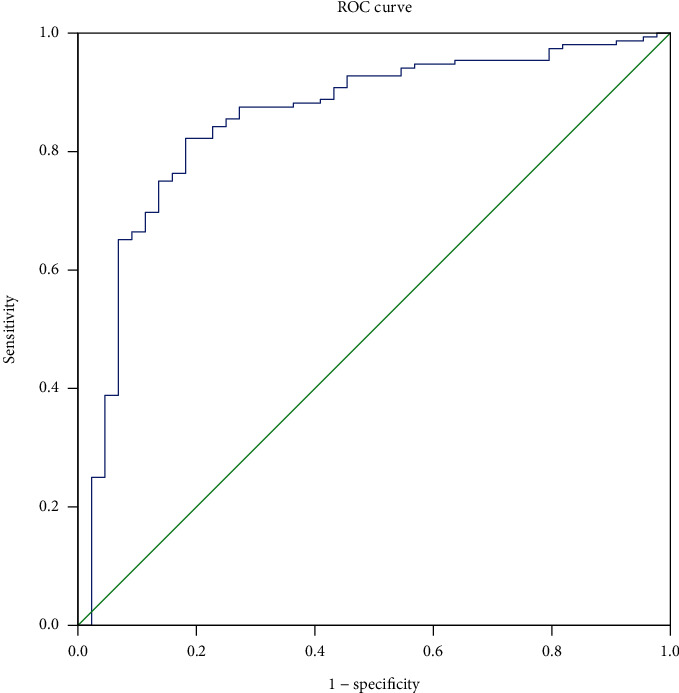
ROC curves for IDO in predicting CKD.

ROC curve was employed to evaluate the prediction efficiency of IDO on the CKD. The area under the ROC curve was 0.825 (95% CI: 0.732–0.919, *p* < 0.001) for IDO. The analysis of ROC curves revealed a best cut­off for IDO was 0.0466 and yielded a sensitivity of 83.8% and a specificity of 75% (Figure 2).

### 4.2. Correlation of IDO and ACR in CKD Patients

A total of 176 patients completed the ACR test. According to the ACR level, patients were divided into the ACR1 (<30 mg/g) group, the ACR2 (30-300 mg/g) group, and the ACR3 (>300 mg/g) group. The age of the ACR3 group was significantly lower than that of the ACR2 group (*p* < 0.05), but there was no significant difference between the ACR3 group and the ACR1 group. Hemoglobin, total protein, and albumin in the ACR1 group were significantly higher than those in the ACR2 group and the ACR3 group (*p* < 0.01), while BUN, sCr, Kyn, low-density lipoprotein, uric acid, and IDO levels were significantly lower than those in the ACR2 group and the ACR3 group (*p* < 0.01 or *p* < 0.05). Fasting blood glucose (*p* < 0.05), retinol-binding protein (*p* < 0.01), and total cholesterol (*p* < 0.05) in the ACR1 group were significantly lower than those in the ACR3 group, while Trp in the ACR3 group was significantly higher than that in the ACR3 group (*p* < 0.01). Complement C4 and CRP were significantly lower in the ACR1 group compared with the ACR2 group (*p* < 0.05), but no significant difference was observed between the ACR3 group and the ACR1 group (Table 4).

The above correlation analysis has indicated that there was a positive correlation between ACR and IDO. Logarithm was performed to transform IDO into ln(IDO) which is normal distribution data. Binary logistic regression showed that ln(IDO) was an independent risk factor of ACR. After adjusting for gender, age, hemoglobin, complement C3, CRP, hypertension, diabetes, coronary artery disease, cerebral infarction, and immunoglobulin G, ln(IDO) was still an independent risk factor for ACR (OR = 2.7, *p* < 0.05) (Table 5).

### 4.3. The Efficiency of IDO to Predict ACR

**Figure 3:**
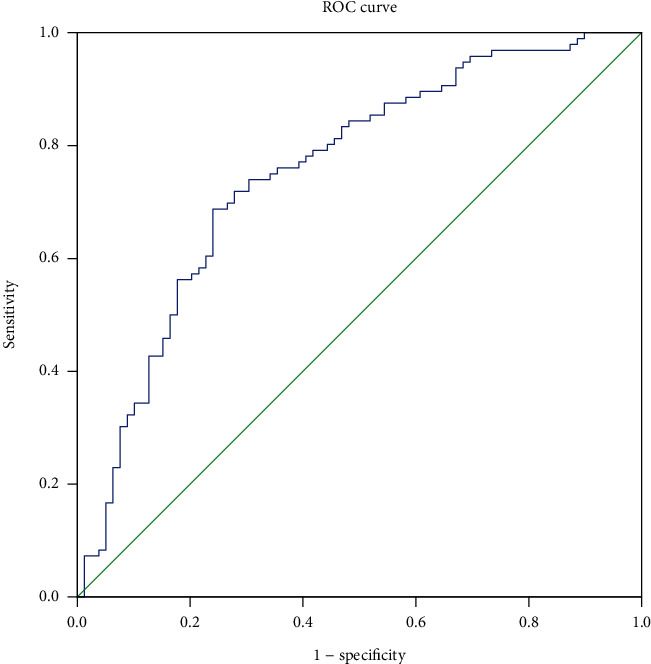
ROC curves for IDO in predicting ACR.

ROC curve was used to evaluate the predictive effect of IDO on ACR. The area under the ROC curve of IDO was 0.753 (95% CI: 0.68-0.826, *p* < 0.001). The analysis of ROC curves revealed a best cut­off for IDO was 0.075 and yielded a sensitivity of 71.9% and a specificity of 72.2% (Figure 3).

## 5. Discussion

Our study compared IDO activity calculated by the ratio of Kyn and Trp between CKD patients and non-CKD patients. The IDO activity showed an intensified trend with significant higher levels in CKD stage 4 and CKD stage 5 compared to other stages. IDO activity was also higher in elevated ACR patients. Furthermore, IDO had a pretty high correlation coefficient with eGFR and ACR which were verified by the binary logistic regression analysis. On top of that, the ROC curve showed that IDO had a rather high ability to predict CKD with 83.8% in sensitivity and 75% in specificity and ACR with 71.9% in sensitivity and 72.2% in specificity suggesting IDO may be a promising biomarker for assessing CKD.

IDO is a rate-limiting enzyme of Trp which is an essential amino acid for the human body. Disorder of Trp metabolism is associated with many diseases. More than 75% of the clear cell in renal cell carcinoma patients contained elevated levels of IDO [15] which was also observed in CKD patients, and IDO activity was correlated with disease severity and levels of inflammatory markers [16]. Moreover, the increment of IDO was also observed in the crescentic glomerulonephritis model which was exacerbated after dealing with 1-methyltryptophan [17] attributed to breaking down the immune tolerance induced by IDO. This kind of immune tolerance is fulfilled via differentiating T cells into regulatory T cells which plays an important role in renal allograft rejection [18–20]. Patients with acute kidney allograft rejection revealed higher expressions of IDO compared with nonrejectors [19]. Although the previous study has proved the IDO activity was an independent risk factor of sCr [16], the accurate correlation between IDO and kidney function is largely unknown. IDO has been suggested to be associated with fibrosis which may be controversial in different fields. In the field of a liver fibrosis model induced by CCl4, mesenchymal stem cells could attenuate liver fibrosis by increasing IDO [19] and deficiency of IDO could enhance the inflammation in the liver and aggravated liver fibrosis [21]. Further, IDO knockout mice also showed more susceptible to high fat diet-induced hepatic inflammation and fibrosis [22]. Another study showed that using 1-methyltryptophan to inhibit IDO could enhance the suppression of activated hepatic stellate cells which mediates the extracellular matrix deposition in the liver to promote fibrosis [23]. Fibrosis in cardiac tissue was also induced by overexpression of IDO [11] via cell arrest. Mutations in the cystic fibrosis have been reported to have a defect in IDO expression and resulted in intensified fibrosis [6]. On the contrary, kidney studies revealed elevated expression of IDO in tubules in the unilateral ureteral obstruction model and transforming growth factor-*β* stimulated MDCK cells [9] as well as diabetic nephropathy patients [10]. Our study also revealed increased activity of IDO in CKD patients, especially in CKD stage 5.

In our study, the correlation coefficient between eGFR and IDO was 0.8, which was higher than that in a previous study [16]. Further, binary logistic regression showed age, IDO, and ACR were independent risk factors for CKD. As is known that age and proteinuria are independent risk factors for CKD [24], while IDO was a novel risk factor of CKD. In addition, previous studies in diabetic patients [16] and obstructive nephropathy [12] have confirmed that IDO is associated with eGFR. However, whether IDO can be used to evaluate the development and progression of CKD still needs a large scale of clinical studies to provide more conclusive evidence.

Renin-angiotensin-aldosterone system inhibitors have been reported to inhibit the activity of Kyn which has a positive correlation with albuminuria [25]. However, this study did not explore the relationship between IDO and albuminuria. In pregnant patients, lower IDO expression level was associated with more severe pregnancy-related hypertension and proteinuria [26]. Therefore, the relationship between IDO and proteinuria is still controversial. Our results suggest that IDO is positively correlated with proteinuria, suggesting that disorder of IDO activity may increase proteinuria. Further binary regression analysis confirmed that IDO was an independent factor of ACR.

In this study, IDO was also found to be associated with other CKD-related indicators, such as calcium, phosphorus, uric acid, hemoglobin, albumin, coagulation indicators, and hypertension. CKD patients, especially those with CKD3 or server stage are commonly accompanying with low calcium, high phosphorus, hyperuricemia, renal anemia, and protein malnutrition as well as increased prevalence of hypertension [27]. Meanwhile, the level of IDO was increased with the increment of the CKD stage. Therefore, correlations between IDO and the above indicators may be caused by CKD itself. The correlation between IDO and coagulation parameters has not been reported before. CKD leads to the accumulation of uremic toxin, which has a toxic effect on blood and vascular wall. Some uremic toxins can be metabolized in the intestinal tract through indole and Kyn pathways. Studies have confirmed that these uremic toxins may be associated with thrombosis in chronic kidney disease by increasing plasma procoagulant factor levels, hyperactivity of platelets, impaired endothelial dysfunction/endothelial healing, decreased nitric oxide bioavailability, and production of procoagulant particles [28]. Therefore, IDO may indirectly affect the coagulation function of CKD patients to promote thrombosis. The specific mechanism needs further research. The correlation between IDO and CRP seems to be similar to previous studies [29], suggesting that IDO may be involved in the chronic inflammatory state of CKD; however, in our study, there was no significant difference in CRP levels among different groups, suggesting that IDO does not promote the progress of CKD by increasing chronic inflammatory level.

There are some limitations in the present study. Firstly, we did not recruit enough CKD stage 1 and CKD stage 2 patients to observe the level of IDO in these two stages which was the same as a previous study [16]. It could be useful to study the levels of IDO in these two stages which could be better for assessing kidney function. Secondly, the sample size in our study was still not large enough although bigger than a previous study [9, 16]. But the correlation coefficient of eGFR and IDO was pretty high, and the ROC showed that IDO was a promising biomarker for CKD enhancing the negative relationship between IDO and CKD.

Taken together, IDO was an independent influence factor of eGFR and ACR and could be used as a biomarker to assess CKD.

## Figures and Tables

**Table 1:** Clinical characteristics of CKD patients and non-CKD patients.

	Non-CKD	CKD3	CKD4	CKD5
Gender (male/female)	19/23	38/16	34/15	25/17
Age (years)	62 ± 13^#▲^	69 ± 16	75 ± 12	71 ± 11
Uric acid (*μ*mol/l)	305 ± 99^∗^^#▲^	391 ± 95	475 ± 141	412 ± 105
Albumin (g/l)	39.9 ± 2.9^∗^^#▲^	36.1 ± 5.8	35.5 ± 5.0	32.9 ± 4.8
Prealbumin (mg/l)	225.1 ± 53.5	229.8 ± 79.7	222.3 ± 68.7	232.3 ± 72.5
FT3 (pmol/l)	4.1 ± 0.9^#▲^	3.7 ± 0.8	3.4 ± 0.7	3.3 ± 1.1
FT4 (pmol/l)	13.2 ± 2.6	12.6 ± 1.9	13.0 ± 2.3	12.3 ± 2.2
TSH (mIU/l)	2.2 ± 1.1	2.0 ± 1.3	2.3 ± 1.4	2.7 ± 1.9
Complement C3 (mg/l)	0.87 ± 0.18^∗^	0.70 ± 0.19	0.79 ± 0.17	0.71 ± 0.19
Complement C4 (mg/l)	0.20 ± 0.06	0.25 ± 0.18	0.21 ± 0.06	0.24 ± 0.08
Hemoglobin (g/l)	130 ± 16^#▲^	122 ± 20	109 ± 25	89 ± 15^∗^
Cystatin C (mg/dl)	1.19 ± 0.36^∗^^#▲^	1.75 ± 0.40	2.65 ± 0.61	3.95 ± 0.71^∗^
*β*2-MG (mg/dl)	2.55 ± 1.10^∗^^#▲^	4.08 ± 1.63	7.32 ± 2.79	12.76 ± 4.33^∗^
BUN (mmol/l)	5.71 ± 1.73^∗^^#▲^	8.43 ± 2.83	14.09 ± 7.42	21.92 ± 5.30^∗^
sCr (*μ*mol/l)	67 ± 14^∗^^#▲^	120 ± 21	191 ± 37	471 ± 152^∗^
Proteinuria (g/24 h)	36 ± 47^∗^^#▲^	555 ± 1124	767 ± 976	1582 ± 1918
Calcium (mmol/l)	2.24 ± 0.13^∗^^#▲^	2.14 ± 0.15	2.17 ± 0.13	2.03 ± 0.21
Phosphorus (mmol/l)	1.18 ± 0.19^#▲^	1.16 ± 0.17	1.30 ± 0.23	1.52 ± 0.36^∗^
TC (mmol/l)	4.35 ± 1.01	4.48 ± 1.63	4.06 ± 1.16	3.97 ± 0.97
TG (mmol/l)	1.87 ± 0.89^▲^	1.61 ± 0.96	1.58 ± 0.71	1.38 ± 0.93
HDL (mmol/l)	1.11 ± 0.29	1.00 ± 0.28	0.96 ± 0.24	1.03 ± 0.51
LDL (mmol/l)	2.25 ± 0.64	2.53 ± 1.25	2.24 ± 0.82	2.20 ± 0.77
HbA1c (%)	7.28 ± 1.71	6.86 ± 1.14	6.45 ± 0.86	6.19 ± 0.92
IgA (mg/l)	2.25 ± 0.89	2.53 ± 2.05	2.58 ± 1.36	2.34 ± 0.92
IgG (mg/l)	10.23 ± 1.96	11.39 ± 4.98	12.15 ± 3.40	11.81 ± 4.76
IgM (mg/l)	1.04 ± 0.53	0.89 ± 0.68	0.87 ± 0.46	0.96 ± 1.05
Tryptophan (*μ*mol/l)	57.3 ± 21.7^▲^	55.9 ± 21.4	48.3 ± 21.6	35.3 ± 17.1^∗^
Kynurenine (*μ*mol/l)	2.15 ± 0.82^∗^^#▲^	3.30 ± 1.38	4.23 ± 2.02	5.34 ± 2.73
IDO	0.04 ± 0.02^∗^^#▲^	0.07 ± 0.04	0.10 ± 0.06	0.16 ± 0.08^∗^
CRP (mg/l)	9 ± 16	12 ± 29	27 ± 38	19 ± 35
PT (second)	11.21 ± 1.00	11.16 ± 1.12	11.79 ± 1.43	12.30 ± 2.79
APTT (second)	26.54 ± 2.20	27.11 ± 2.89	27.22 ± 3.47	30.71 ± 10.54
Fibrinogen (g/l)	3.35 ± 1.04	3.22 ± 0.72	3.92 ± 1.32	4.97 ± 3.82
DD (*μ*g/ml)	0.57 ± 0.48	1.30 ± 2.61	2.12 ± 5.08	1.14 ± 0.82

CKD: chronic kidney disease; FT3: free triiodothyronine; FT4: free thyroxine; TSH: thyroid-stimulating hormone; *β*2-MG: *β*2-microglobulin; BUN: blood urea nitrogen; sCr: serum creatinine; TC: total cholesterol; TG: triglyceride; HDL: high-density lipoprotein; LDL: low-density lipoprotein; IgA: immunoglobulin A; IgG: immunoglobulin G; IgM: immunoglobulin M; IDO: indoleamine-2,3-dioxygenase; CRP: C-reactive protein; PT: prothrombin time; APTT: activated partial thromboplastin time; DD: D-dimer. ^∗^*p*, non-CKD vs. CKD3; ^#^*p*, non-CKD vs. CKD4; ^▲^*p*, non-CKD vs. CKD5; ^∗^^#▲^*p* < 0.05.

**Table 2:** Correlations of IDO and CKD-related indices.

	*r*	*p*
eGFR	-0.8	<0.01^#^
Age	0.3	<0.01^#^
Albumin	-0.6	<0.01^#^
Ca	-0.5	<0.01^#^
P	0.4	<0.01^#^
Hb	-0.6	<0.01^#^
CRP	0.3	<0.01^#^
Proteinuria	0.4	<0.01^#^
ACR	0.5	<0.01^#^
PT	0.3	<0.01^#^
APTT	0.2	<0.01^#^
FN	0.4	<0.01^#^
DD	0.4	<0.01^#^
IgA	0.1	0.1
IgG	0.2	0.05
IgM	0.1	0.5
Complement C3	-0.1	0.3
Complement C4	0.1	0.2
HP	0.2	<0.01^#^
DM	0.1	0.2
CAD	0.04	0.6
CI	0.1	0.4
Gender	0.03	0.7
UA	0.4	<0.01^#^
TC	-0.1	0.19
LDL	-0.01	0.9
HbA1c	-0.2	0.1

eGFR: estimated glomerular filtration rate; Ca: serum calcium; P: serum phosphorus; Hb: hemoglobin; CRP: C-reactive protein; ACR: albuminuria and creatinine ratio; PT: prothrombin time; APTT: activated partial thromboplastin time; FN: fibrinogen; DD: D-dimer; IgA: immunoglobulin A; IgG: immunoglobulin G; IgM: immunoglobulin M; HP: hypertension; DM: diabetes mellitus; CAD: coronary artery disease; CI: cerebral infarction; UA: uric acid; TC: total cholesterol; LDL: low-density lipoprotein. ^#^*p* < 0.01.

**Table 3:** Binary logistic analysis of independent risk factors of chronic kidney disease.

	Exp(B) (95% CI)	*p*
Age	1.07 (1.01-1.13)	0.018^∗^
Hypertension	1.14 (0.32-4.04)	0.85
Diabetes	3.88 (0.90-16.8)	0.07
Coronary artery disease	0.61 (0.16-2.38)	0.48
Cerebral infarction	2.47 (0.55-11.1)	0.24
ln(IDO)	3.48 (1.16-10.47)	0.027^∗^
Albuminuria & creatinine ratio	1.14 (1.05-1.24)	0.002^#^

IDO: indoleamine-2,3-dioxygenase; ^∗^*p* < 0.05, ^#^*p* < 0.01.

**Table 4:** Comparison of clinical indices among different ACR groups.

	ACR1 (<30 mg/g)	ACR2 (30-300 mg/g)	ACR3 (>300 mg/g)
Age (years)	68.76 ± 14.58	71.14 ± 12.04	64.38 ± 16.72^∗^(3 vs. 2)
Gender (male/female)	47/33	45/25	17/9
Hemoglobin (g/l)	121.3 ± 20.4^#^	108.3 ± 25.6	102.2 ± 27.9
Total protein (g/l)	65.0 ± 7.29^#^	60.1 ± 7.05	61.7 ± 11.9
Albumin (g/l)	38.2 ± 4.0^#^	34.4 ± 5.0	33.0 ± 6.9
Prealbumin (mg/l)	224.2 ± 70.6	227.6 ± 64.3	244.8 ± 75.3
Cystatin C (mg/dl)	4.17 ± 1.13	2.87 ± 1.05	3.09 ± 1.24
BUN (mmol/l)	8.46 ± 4.59^#^	15.2 ± 8.27	15.6 ± 8.8
sCr (*μ*mol/l)	124.9 ± 109.4^#^	247.4 ± 159.3	309.9 ± 207.8
FBG (mmol/l)	5.84 ± 2.33^∗^ (3 vs. 1)	5.48 ± 2.26	4.83 ± 1.47
Uric acid (*μ*mol/l)	370.6 ± 132^∗^	425.2 ± 123.5	406.1 ± 106.6
RBP (mg/l)	20.6 ± 31.9^#^ (3 vs. 1)	43.8 ± 43.3	89.3 ± 36.0
TC (mmol/l)	4.04 ± 0.97^∗^ (3 vs. 1)	4.36 ± 1.37	4.65 ± 1.63
TG (mmol/l)	1.84 ± 1.56	1.59 ± 0.85	1.55 ± 0.76
LDL (mmol/l)	2.1 ± 0.64^∗^	2.49 ± 1.01	2.63 ± 1.27
HDL (mmol/l)	1.05 ± 0.3	0.97 ± 0.25	1.1 ± 0.34
HbA1c (%)	11.17 ± 30.7	6.72 ± 1.06	6.66 ± 0.82
IgG (mg/l)	12.25 ± 3.11	10.92 ± 3.90	12.56 ± 7.05
IgA (mg/l)	2.40 ± 1.43	3.86 ± 1.23	2.68 ± 1.97
IgM (mg/l)	0.92 ± 0.57	1.25 ± 2.06	0.97 ± 0.78
Complement C3 (mg/l)	0.77 ± 0.16	0.74 ± 0.19	0.71 ± 0.23
Complement C4 (mg/l)	0.20 ± 0.06^∗^ (2 vs. 1)	0.25 ± 0.15	0.23 ± 0.11
CRP (mg/l)	9.26 ± 12.94^∗^ (2 vs. 1)	23.73 ± 40.79	22.12 ± 40.33
Tryptophan (*μ*mol/l)	54.30 ± 20.23^#^ (3 vs. 1)	49.00 ± 23.67	43.36 ± 23.73
Kynurenine (*μ*mol/l)	3.00 ± 1.42^#^	4.46 ± 2.45	4.50 ± 2.17
IDO	0.072 ± 0.092^∗^	0.107 ± 0.071	0.126 ± 0.075

ACR: albuminuria and creatinine ratio; BUN: blood urea nitrogen; sCr: serum creatinine; FBG: fast blood glucose; RBP: retinol-binding protein; TC: total cholesterol; TG: triglyceride; LDL: low-density lipoprotein; HDL: high-density lipoprotein; IgG: immunoglobulin G; IgA: immunoglobulin A; IgM: immunoglobulin M; CRP: C-reactive protein; IDO: indoleamine-2,3-dioxygenase. ^∗^*p* < 0.05, ^#^*p* < 0.01.

**Table 5:** Binary logistic analysis of independent risk factors of ACR.

	Exp(B) (95% CI)	*p*
Gender	1.24 (0.5-3.2)	0.66
Age	0.97 (0.94-1.01)	0.15
Hemoglobin	0.99 (0.97-1.01)	0.57
Complement C3	0.26 (0.02-3.58)	0.32
C-reactive protein	1.01 (0.99-1.04)	0.08
Hypertension	0.66 (0.23-1.89)	0.44
Diabetes mellitus	0.41 (0.16-1.03)	0.06
Coronary artery disease	1.78 (0.69-4.59)	0.24
Cerebral infarction	0.67 (0.25-1.80)	0.43
ln(IDO)	2.70 (1.20-6.10)	0.02^∗^
Immunoglobulin G	0.90 (0.81-1.01)	0.07

IDO: indoleamine-2,3-dioxygenase; ^∗^*p* < 0.05.

## Data Availability

The data can be available via sending email to panbinbin@njmu.edu.cn or wanxin@njmu.edu.cn.
